# Key factors influencing patient satisfaction in the emergency department of a tertiary hospital in Saudi Arabia: a cross-sectional study

**DOI:** 10.1186/s12913-026-15082-0

**Published:** 2026-07-10

**Authors:** Kholoud Abdullah Babkair, Sawsan Hanafi, Mohamed Eldigire Ahmed, Mahmoud Ahmad Wazzan, Khaled Fatani, Mohammad Alharbi, Ammar Mashat, Loai Alsulaimani, Hotoun Bokhari

**Affiliations:** 1 Emergency Department, King Abdulaziz Medical City, Ministry of National Guard Health Affairs, Jeddah, Saudi Arabia; 2 King Abdullah International Medical Research Center, Jeddah, Saudi Arabia; 3 College of Medicine, King Saud bin Abdulaziz University for Health Sciences, Jeddah, Saudi Arabia; 4 Basic Science Department, College of Science and Health Professions, King Saud bin Abdulaziz University for Health Sciences, Jeddah, Saudi Arabia

**Keywords:** Emergency department, Patient satisfaction, Saudi Arabia, Questionnaire, Communication skills, Waiting time

## Abstract

**Background:**

Patient satisfaction is a key indicator of healthcare quality and an essential measure of emergency department (ED) performance. In high-demand ED environments, satisfaction reflects not only clinical outcomes, but also communication, waiting time, clarity of processes, and the overall patient experience. The aim of this study was to identify clinical and non-clinical factors that influence patient satisfaction in the ED of a tertiary healthcare center in Saudi Arabia.

**Methods:**

This analytical cross-sectional study included Arabic or English speaking adult patients (≥ 18 years) attending the ED of a tertiary care center in Saudi Arabia after triage and clinical stabilization. Patients triaged as levels I–II, arriving by ambulance, medically unstable, or having impaired cognitive or mental status were excluded. The target sample size was estimated using a 95% confidence level, 5% margin of error, and 50% response distribution, which yielded an approximate target range of 280–380 participants for a large ED population; 298 complete questionnaires were included. A structured questionnaire covering demographics, 21 satisfaction factors, and three global satisfaction items was administered. Descriptive statistics, Spearman correlation, and multivariable linear regression were used; significance was set at *p* < 0.05.

**Results:**

A total of 298 patients were included, and the response rate was 75.06% of eligible participants (298/397). Most participants reported generally positive experiences across satisfaction domains. Nearly all factors showed significant positive correlations with overall satisfaction, and the strongest associations were observed for communication with physicians (*ρ =* 0.46), communication with nurses (*ρ =* 0.40), explanation of the treatment provided (*ρ =* 0.38), and waiting time during stay (*ρ =* 0.41). In the multivariable model (*R*² *=* 0.50), four factors remained significant predictors: communication with physicians, communication with nurses, explanation of the treatment provided, and waiting time during stay. Environmental and facility-related factors were less influential after adjustment.

**Conclusions:**

Communication and timely care play central roles in shaping patient satisfaction in the ED. These findings highlight the value of improving communication skills among healthcare providers and workflow processes to reduce waiting times. Future studies should incorporate length of stay and examine how different clinical presentations influence satisfaction within the ED environment.

**Supplementary Information:**

The online version contains supplementary material available at 10.1186/s12913-026-15082-0.

## Background

Patient satisfaction refers to a patient’s perception of the care that they have received and how well it aligns with their expectations. Patient satisfaction has become a cornerstone of healthcare delivery and a key indicator of service quality in various medical settings. With increasing emphasis on patient-centered care, understanding patients’ experiences, expectations, and perceptions has become essential for evaluating healthcare systems. The emergency department (ED) is often the first point of contact for individuals seeking urgent medical attention and represents a unique environment for assessing satisfaction because of its fast-paced, high-pressure, and unpredictable nature [1, 2]. Multiple challenges inherent to the ED make achieving high satisfaction levels particularly difficult, such as long waiting times, overcrowding, communication gaps, and patients’ physical or emotional distress [3–6]. Nevertheless, measuring satisfaction in this setting is important as it is linked to clinical outcomes, adherence to medical advice, and patients’ overall perception of healthcare quality.

Emergency departments carry a substantial international service burden. Recent population-based data from high-income systems show wide variation in annual ED use, ranging from 248.4 to 443.2 visits per 1,000 population/year across New Zealand, New York, and Ontario [7]. In the United States, approximately 155 million ED visits occurred in 2022, equivalent to 47 visits per 100 persons [8]. In Saudi Arabia, Ministry of Health hospitals recorded 14,338,219 medical emergency encounters and 2,552,209 surgical emergency encounters in 2023, with Jeddah reporting 775,037 medical and 27,660 surgical emergency encounters in Ministry of Health hospitals during the same year [9]. At the study hospital, the ED receives approximately 220 patients per day, corresponding to nearly 80,000 visits annually according to institutional operational data. Patients visit EDs for a wide range of acute medical and surgical problems, including trauma and injuries, fever and infection-related complaints, respiratory symptoms, chest pain and other cardiovascular presentations, abdominal pain and gastrointestinal symptoms, neurologic complaints, and pain-related conditions. This high volume and broad case-mix make the ED a particularly important setting for evaluating patient satisfaction, because patients’ perceptions are shaped by both the clinical encounter and the surrounding service process.

Multiple clinical and non-clinical factors shape how patients perceive their ED experience. Interpersonal interactions, particularly effective communication between healthcare providers and patients, have consistently been identified as strong predictors of satisfaction. Patients value clear explanations of their condition, treatment plans, and expectations for recovery. Empathy, professionalism, and provider attitudes have also been shown to significantly enhance satisfaction. In contrast, several studies report that longer waiting times negatively influence satisfaction and may be the primary determinant of dissatisfaction in the ED in some cases [10, 11]. Beyond the ED, research in broader hospital populations has shown that patient-reported experiences and fulfillment of expectations are also closely related to overall satisfaction [12]. Similar patterns have been reported internationally, where provider behavior and communication quality were found to be major drivers of satisfaction in emergency departments [6, 13].

In Saudi Arabia, limited research has explored the specific factors influencing patient satisfaction in ED settings. Available studies in this context have reported varying findings, with some identifying empathy as the primary driver of satisfaction, while others emphasize waiting time, communication, or perceived health improvement [14, 15]. This variability highlights the need for context-specific evidence from Saudi tertiary care centers.

Therefore, the aim of this study was to identify and rank the factors that influence patient satisfaction in the emergency department of a tertiary healthcare center. Both clinical and non-clinical aspects of care and their association with patient demographics were considered. The findings could contribute meaningful insights to improve patient experience and advance the goals of patient-centered healthcare.

## Methods

### Study design and setting

This analytical cross-sectional study was conducted in the ED of a tertiary hospital in Jeddah, Saudi Arabia. This ED provides 24-hour acute care service and serves as a major referral center in the western region. Data were collected using a questionnaire across multiple morning, afternoon, and night shifts between April 18, 2024, and December 14, 2024.

### Participants, eligibility criteria, and response rate

All adult patients who were physically present in the ED during data-collection shifts were screened for eligibility. A total of 836 patients were present during these periods. Patients were excluded if they were triaged as level I or II, arrived by ambulance, were medically unstable, required immediate clinical intervention, or had impaired cognitive or mental status that limited their ability to understand and respond to the questionnaire. These exclusions were applied to avoid interfering with urgent care and to protect patients who were clinically or emotionally unsuitable for participation.

**Fig. 1:**
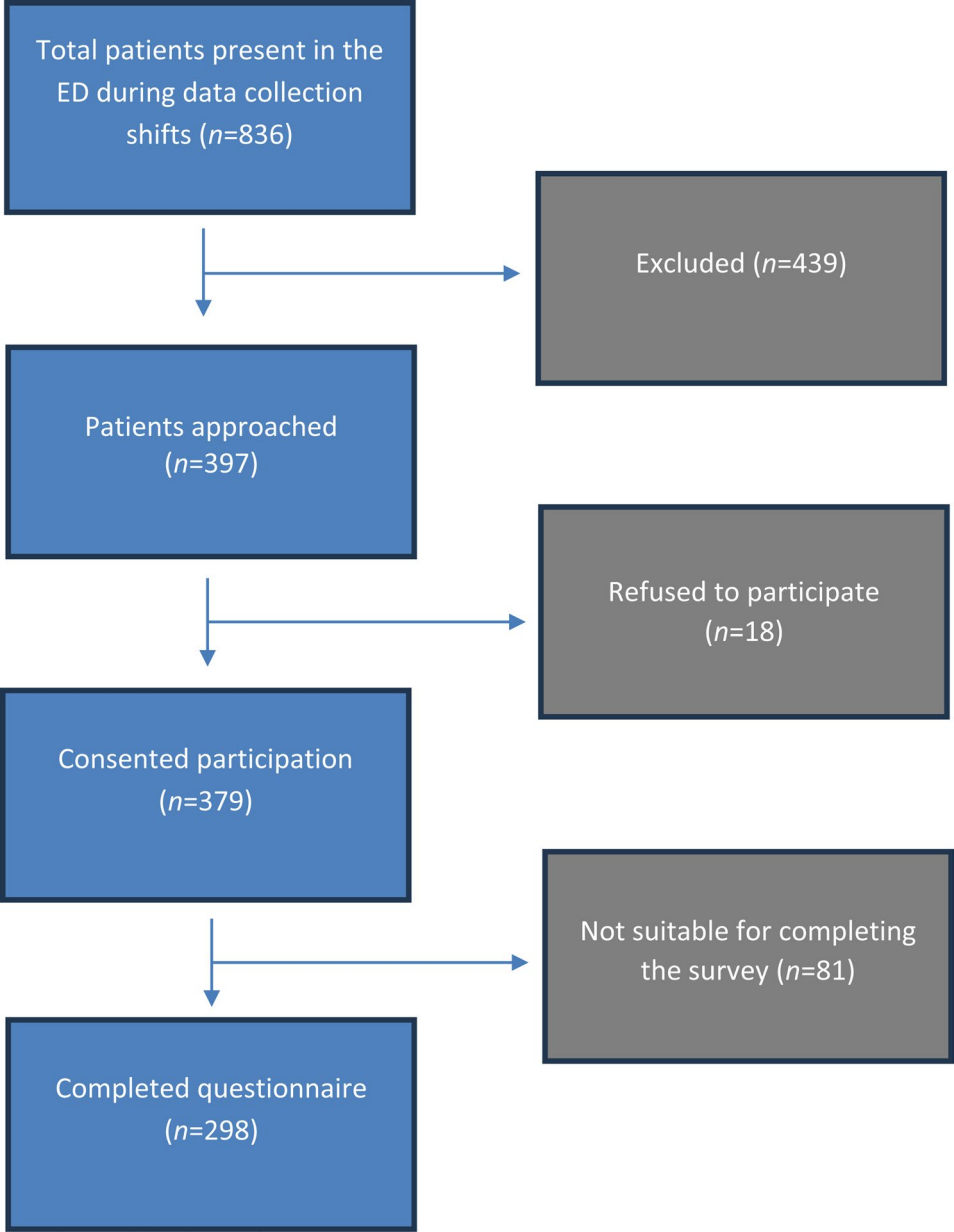
Study flow diagram

The remaining eligible patients were approached after triage and clinical stabilization. Of those, 18 refused participation, and 379 provided written informed consent. For illiterate patients who agreed to participate, the questionnaire was administered with the assistance of their companions, who read the items aloud exactly as written and recorded the patients’ responses. In addition, if a consenting patient became distressed, angry, tearful, or otherwise unsuitable for continuing during questionnaire completion, the survey was stopped and the partial response was excluded. This resulted in a final sample of 298 fully completed questionnaires being included in the analysis. The response rate was defined as the proportion of completed surveys among eligible patients who were approached, which was 75.06% (298/397). For transparency, the crude completion percentage of all ED patients present during the data-collection shifts, including those who were ineligible or not approached, was 35.65% (298/836) (Fig. 1).

### Sampling method

A non-probability convenience-sampling approach was used. We acknowledge that probability sampling is generally less prone to selection bias; however, a complete real-time sampling frame and random selection of ED attendees were not operationally feasible in this acute clinical setting because patient flow, acuity, waiting time, discharge timing, and suitability for participation varied continuously across shifts. To improve the heterogeneity of the sample and reduce selection bias as far as possible, data were collected across morning, afternoon, and night shifts over the study period, and eligible patients were approached after triage and clinical stabilization. The use of non-probability sampling was considered in the interpretation of the findings and is acknowledged as a limitation. To estimate an approximate target sample, the Raosoft^®^ online sample-size calculator was consulted using a 95% confidence interval (CI), 5% margin of error, and 50% response distribution. For a large ED population, these parameters typically yield a recommended sample in the range of approximately 280–380 participants. Data collection continued across multiple shifts until 298 complete responses were obtained. This sample size falls within the recommended range and is adequate for the purposes of the study, including correlation analyses and multivariable regression.

### Data collection procedure

The research team visited the ED during different shifts throughout the data-collection period and approached patients after triage and clinical stabilization. The purpose of the study was explained, written informed consent was obtained, and the questionnaire was administered in either Arabic or English. For patients who appeared distressed or unsuitable for continuation of the questionnaire, data collection was immediately stopped out of respect for their comfort, and their partial responses were excluded. No compensation was provided.

### Study tool

A structured questionnaire was developed for this study after reviewing relevant ED patient-satisfaction and patient-experience literature, including studies and reviews addressing provider communication, empathy and attitude, waiting time, treatment explanation, perceived improvement, discharge information, administrative processes, environmental comfort, privacy, overall satisfaction, and recommendation of care [1–3, 5, 6, 16–21]. The content of the questionnaire was then refined through expert consultation. The first section focused on demographic characteristics (5 items) and collected information on age, sex, educational level, marital status, and knowledge of the ED triage system. The second section focused on satisfaction factors (21 items), and the items assessed satisfaction with multiple aspects of the ED experience, including road signage and parking, reception and registration processes, comfort and cleanliness of the waiting area, availability of amenities, communication with physicians and nurses, explanation of treatment, perceived improvement, and privacy. Each item was rated on a 5-point Likert scale (1 = very dissatisfied, 5 = very satisfied).

The third section concerned global satisfaction (3 items) and examined the perception of whether the care met patient expectations, overall satisfaction with the visit, and likelihood of recommending the ED to others. Patient satisfaction was operationalized as a multidimensional patient-reported construct. The 21 satisfaction-factor items were organized conceptually into domains that reflected access and arrival to the ED, administrative and registration processes, waiting-area environment and timeliness, provider communication and clinical-care perceptions, privacy, and discharge-related information. Each item was scored on a 5-point Likert scale, where 1 indicated “very dissatisfied” and 5 indicated “very satisfied”; therefore, higher scores indicated greater satisfaction. No items were reverse coded. The three global satisfaction items assessed whether care met the patient’s expectations, the patient’s overall satisfaction with the ED visit, and whether the patient would recommend the ED to family and friends. These three items were averaged to create the overall satisfaction composite score, which served as the primary outcome in correlation and regression analyses. The questionnaire was reviewed by two emergency physicians and two medical education experts for content and face validity. For linguistic accuracy, it was translated and back-translated successfully by two different faculty in medical education at King Saud bin Abdulaziz University for Health Sciences. Pilot testing (*n =* 32) demonstrated acceptable reliability (Cronbach’s alpha = 0.797). The full questionnaire is provided as Supplementary File 1.

### Data analysis

Data were analyzed using JMP 19 (SAS Institute Inc., Cary, NC, USA). Descriptive statistics were used to summarize patient characteristics and satisfaction items. Continuous variables were reported as means and standard deviations, while categorical variables were presented as frequencies and percentages. Likert-scale satisfaction items were treated as continuous for descriptive purposes, which is consistent with common practice. The primary outcome, overall patient satisfaction, was calculated as the mean of the three global satisfaction items (meeting expectation, overall impression, and recommendation to family and friends).

Because item scores were ordinal and not normally distributed, Spearman’s rank correlation coefficient (*ρ*) was used to examine the bivariate association between each satisfaction factor and the overall satisfaction score. To identify independent predictors of overall satisfaction, a multivariable linear regression model was constructed and included all satisfaction factors and demographic variables. Multicollinearity was assessed using the variance inflation factor (VIF). Regression assumptions (linearity, homoscedasticity, and normality of residuals) were evaluated using residual plots and studentized residual diagnostics. Regression results were reported as beta coefficients (*β*), standard errors, *p*-values, and 95% CIs. A two-sided *p*-value < 0.05 was considered statistically significant.

### Ethical considerations

This study was reviewed and approved by the Institutional Review Board of King Abdullah International Medical Research Center (KAIMARC), Jeddah, Saudi Arabia (approval number: IRB/0554/24; March 26, 2024). All participants were informed about the purpose of the study, and prior to data collection, written informed consent was obtained through an electronic consent form that was included in the questionnaire prior to data collection. Data were collected only after obtaining verbal operational clearance from the supervising attending physician on duty for each shift of the ED. No identifiable personal information was collected, and participation was entirely voluntary. The study adhered to the ethical principles outlined in the Declaration of Helsinki.

## Results

### Patient characteristics

The demographic characteristics of participants are presented in Table 1. The mean age of the sample was 52.2 ± 19.4 years, with participants ranging from young adults to elderly individuals. Females represented 56% of the sample, and most respondents were married (68%). Their educational levels varied, with most participants having completed secondary school or higher. Notably, more than half of the participants (52.3%) reported having no prior knowledge of the ED triage system, and only 15.1% stated they knew what triage is.

**Table 1:** Demographic characteristics of study participants (*N =* 298)

Characteristic	Frequency (*n =* 298)	Percentage (%)
**Age** 52.2 ± 19.4 years		
**Sex**		
Male	131	44
Female	167	56
**Education**		
Illiterate	64	21.5
Less than secondary	67	22.5
Secondary	79	26.5
Bachelor	82	27.5
Postgraduate	6	2
**Marital Status**		
Single	43	14.5
Married	202	68
Divorced	12	4
Widowed	40	13.5
**Patient Knowledge About Triage**		
Do not know anything about triage	156	52.3
Some information about triage	63	21.1
Knows what triage is	79	26.6

### Descriptive statistics of satisfaction items

Overall, the mean scores of the 21 satisfaction factors indicated generally positive patient experiences in the ED (Table 2). The highest-rated items included privacy during stay (4.75 ± 0.69), attitude and communication skills of doctors (4.72 ± 0.83), and attitude and communication skills of nurses (4.70 ± 0.81). Environmental factors such as ventilation, bathroom availability, and cleanliness also received relatively high ratings, whereas operational items such as waiting time during stay (3.40 ± 1.55) and food and beverage availability (3.90 ± 1.25) received more moderate scores. Among the global satisfaction items, the mean score for overall satisfaction was 4.24 ± 1.12, and expectation prior to visit (4.10 ± 1.20) and likelihood of recommending the ED (4.29 ± 1.09) also indicated positive impressions.

**Table 2:** Descriptive statistics of patient satisfaction factors and overall satisfaction items

Item	Mean	Standard Deviation
Non-clinical		
*Mode of arrival to the emergency department*		
Road directions to emergency department	4.64	0.7450
Road Directions to parking slots allocated to emergency department patients	4.43	0.9196
Availability of parking spots	3.68	1.3010
*Administrative skills*		
Check-in procedure by administrative staff	4.70	0.7340
Administrative staff’s attitude	4.67	0.7859
Estimated waiting time for administrative staff	3.27	1.0346
*Reception/waiting area*		
Seats in waiting rooms	4.17	1.1810
Availability of food and beverages in waiting room	3.90	1.2582
Availability of Wi-Fi in the waiting room	3.16	0.8573
Availability of restrooms in the waiting room	4.16	1.2203
Ventilation/AC in waiting room	4.27	1.1372
Cleanliness of waiting room	4.43	0.9727
Time-delay updates given by administrative staff	3.18	0.9056
Waiting time during stay	3.40	1.5523
Clinical		
*Provider’s Interpersonal skills*		
Attitude and communication skills of doctors	4.72	0.8317
Attitude and communication skills of nurses	4.70	0.8091
*Intervention*		
Explanation and treatment	4.62	0.8687
Improvement of condition	3.97	1.1915
Provided information after discharge	3.69	0.9799
Treatment provided for the patient	4.02	1.2016
Privacy during stay	4.75	0.6930
Overall satisfaction		
*Global satisfaction items*		
**Expectation**	**4.10**	1.1973
**Overall**	**4.24**	1.1178
**Recommendation**	**4.29**	1.0874

### Correlations between satisfaction factors and overall satisfaction

The Spearman correlation analysis revealed that almost all satisfaction factors were positively associated with overall satisfaction (Table 3). The strongest correlations were observed for interpersonal communication factors:

**Table 3:** Spearman’s correlation between patient satisfaction factors and overall satisfaction score

Factor	Spearman’s Coefficient	*p*-value
Non-clinical		
*Mode of arrival to the emergency department*		
Road directions to emergency department	0.2495	< 0.0001*
Road directions to parking slots allocated to emergency department patients	0.0937	0.1063
Availability of parking spots	0.1824	0.0016*
*Administrative skills*		
Check-in procedure by administrative staff	0.2905	< 0.0001*
Administrative staff’s attitude	0.3053	< 0.0001*
Estimated waiting time by administrative staff	0.2462	< 0.0001*
*Reception/waiting area*		
Seats in waiting rooms	0.2054	0.0004*
Availability of food and beverages in waiting room	0.1180	0.0417*
Availability of Wi-Fi in the waiting room	-0.0241	0.6781
Availability of restrooms in the waiting room	0.1712	0.0030*
Ventilation/AC in waiting room	0.1816	0.0016*
Cleanliness of waiting room	0.1655	0.0042*
Time-delay updates given by administrative staff	0.2165	0.0002*
Waiting time during stay	0.4061	< 0.0001*
Clinical		
*provider’s Interpersonal skills*		
Attitude and communication skills of doctors	0.4607	< 0.0001*
Attitude and communication skills of nurses	0.4047	< 0.0001*
*Intervention*		
Explanation and treatment	0.3835	< 0.0001*
Improvement of condition	0.2504	< 0.0001*
Provided information after discharge	0.1857	0.0013*
Treatment provided for the patient	0.2705	< 0.0001*
Privacy during stay	0.2001	0.0005*

- Attitude and communication skills of doctors (*ρ* = 0.4607, *p* < 0.0001).
- Attitude and communication skills of nurses (*ρ* = 0.4047, *p* < 0.0001).
- Explanation of treatment provided (*ρ* = 0.3835, *p* < 0.0001).
- Waiting time during stay (*ρ* = 0.4061, *p* < 0.0001).

Environmental and comfort-related factors demonstrated weaker but still significant associations, including cleanliness (*ρ =* 0.1655, *p <* 0.0042), restroom availability (*ρ =* 0.1712, *p <* 0.0030), and availability of comfortable seats in the waiting rooms (*ρ =* 0.2054, *p <* 0.0004).

### Multivariate linear regression analysis

**Fig. 2:**
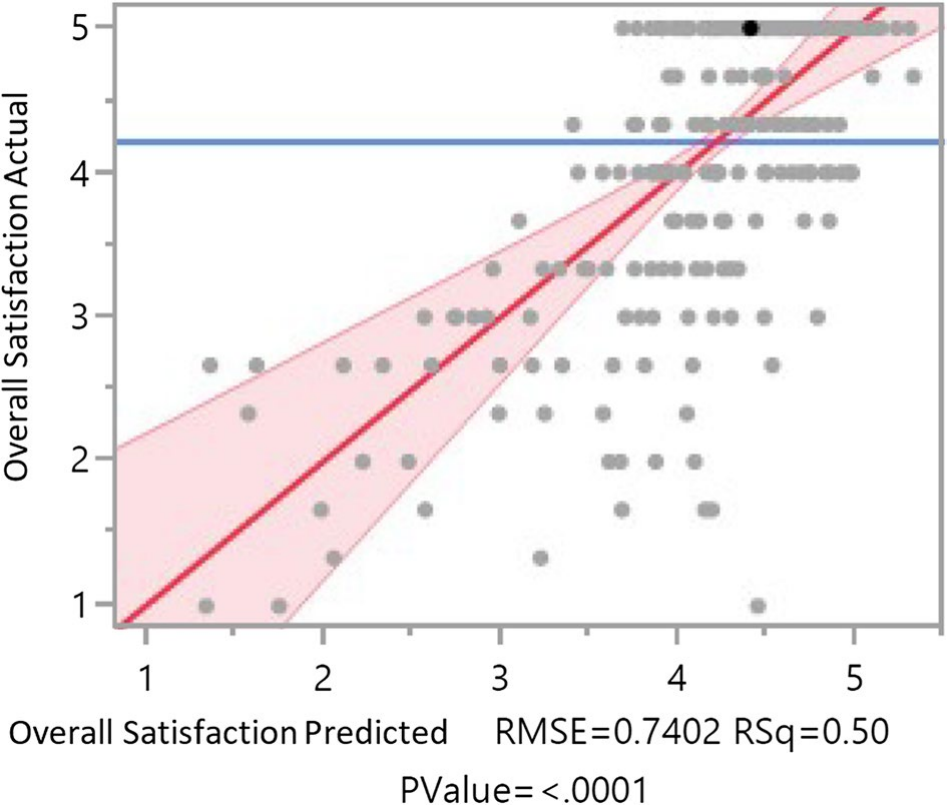
Actual vs. predicted overall satisfaction

To identify independent predictors of overall satisfaction, all satisfaction factors and demographic variables were entered into a multivariable linear regression model. The final model was statistically significant (*p <* 0.0001) and explained approximately 50% of the variance in overall satisfaction (*R*^2^ = 0.50) (Fig. 2). After adjusting for all covariates, four variables remained independent predictors of overall satisfaction (Table 4):

**Table 4:** Multiple linear regression analysis of factors associated with overall satisfaction (*n =* 298)

Predictor	Estimate (β)	Standard Error	t-value	*p*-value	95% CI (Lower-Higher)
Age	0.004	0.003	1.30	0.1946	-0.002 to 0.010
Sex	0.011	0.098	0.11	0.9147	-0.183 to 0.204
Education level	0.035	0.047	0.76	0.4505	-0.057 to 0.127
Marital status	-0.124	0.069	-1.80	0.0723	-0.257 to 0.011
Patient knowledge about triage	-0.006	0.056	-0.10	0.9212	-0.116 to 0.105
Road directions to emergency department	0.131	0.067	1.95	0.0521	-0.001 to 0.263
Availability of parking spots	0.046	0.036	1.34	0.1814	-0.022 to 0.118
Check-in procedure by administrative staff	0.129	0.120	1.07	0.2837	-0.107 to 0.365
Administrative staff’s attitude	-0.056	0.115	-0.50	0.6161	-0.284 to 0.169
Estimated waiting time by administrative staff	0.036	0.053	0.68	0.4977	-0.068 to 0.139
Seats in waiting rooms	0.024	0.054	0.45	0.6521	-0.081 to 0.129
Availability of food and beverages in waiting room	-0.067	0.048	-1.39	0.1661	-0.163 to 0.028
Availability of restrooms in the waiting room	0.002	0.056	0.03	0.9752	-0.109 to 0.112
Ventilation/AC in waiting room	0.038	0.058	0.65	0.5178	-0.077 to 0.152
Cleanliness of waiting room	0.049	0.071	0.69	0.4938	-0.091 to 0.189
Time-delay updates given by administrative staff	0.008	0.058	0.14	0.8870	-0.107 to 0.123
Waiting time during stay	0.151	0.034	4.42	< 0.0001*	0.084 to 0.218
Attitude and communication skills of doctors	0.305	0.074	4.12	< 0.0001*	0.159 to 0.451
Attitude and communication skills of nurses	0.252	0.068	3.71	0.0003*	0.118 to 0.385
Explanation and treatment	0.176	0.062	2.82	0.0051*	0.053 to 0.298
Improvement of condition	0.042	0.103	0.41	0.6834	-0.161 to 0.245
Provided information after discharge	0.054	0.052	1.05	0.2969	-0.048 to 0.156
Treatment provided for the patient	0.027	0.103	0.26	0.7935	-0.177 to 0.231
Privacy during stay	-0.090	0.067	-1.35	0.1785	-0.221 to 0.041

1. Attitude and communication skills of doctors (β = 0.305, *p <* 0.0001).
2. Attitude and communication skills of nurses (β = 0.252, *p <* 0.0003).
3. Explanation of the treatment provided (β = 0.176, *p <* 0.0051).
4. Waiting time during stay (β = 0.151, *p <* 0.0001).

These four factors also demonstrated the strongest relative importance in the effect summary (LogWorth) plot (Table 5), which confirmed that interpersonal communication and waiting experience were the primary drivers of patient satisfaction in the ED. All other service and environmental factors (e.g., cleanliness, ventilation, and food availability) were not significant predictors in the adjusted model, although many showed significant bivariate correlations. There was no evidence of problematic multicollinearity, and all estimated VIF values were less than 4. The regression assumptions were met based on examination of the residual and studentized residual plots.

**Table 5:**
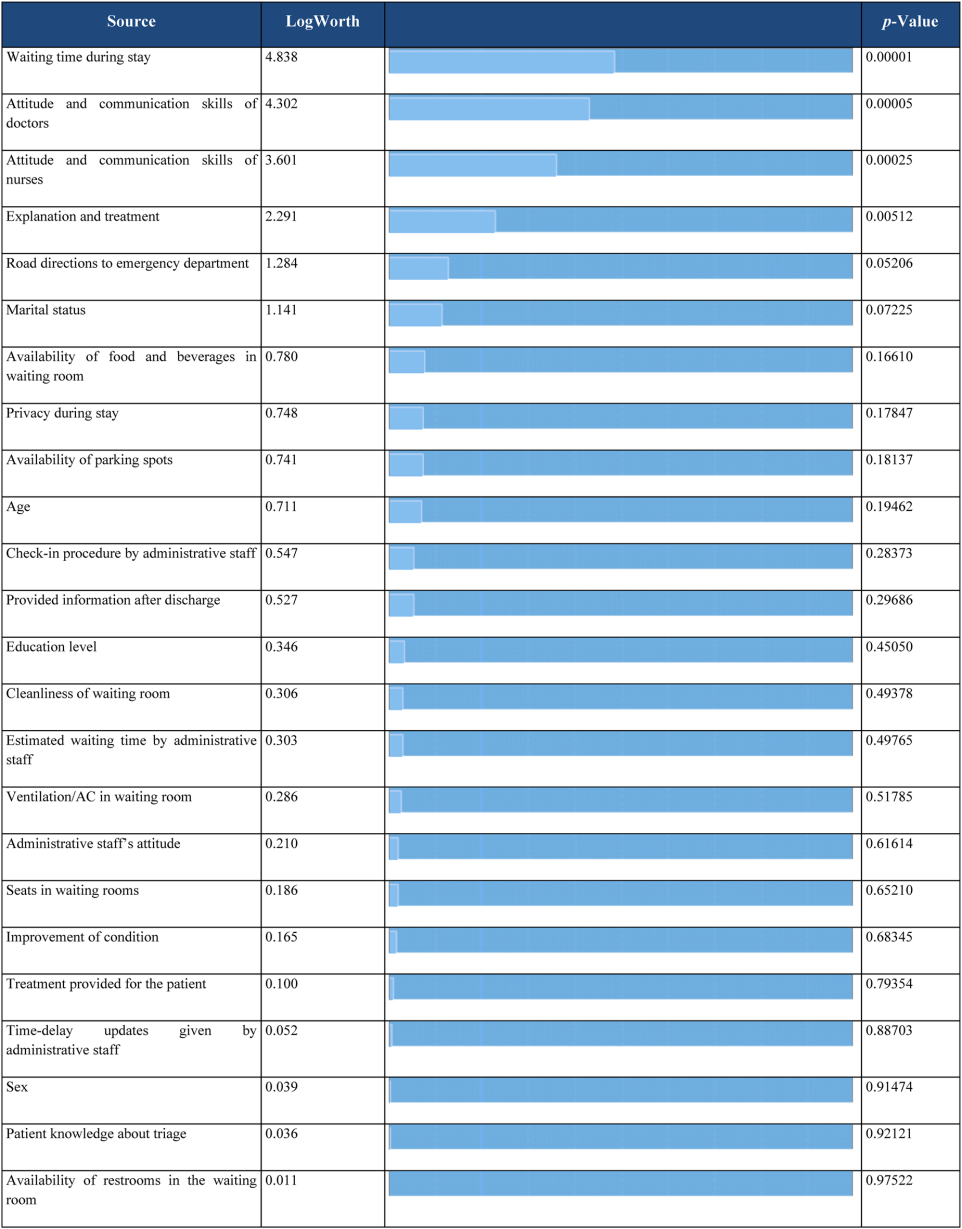
Effect summary (LogWorth) plot

## Discussion

This study found that communication quality and waiting time were the main determinants of overall satisfaction in the ED. The findings are consistent with Saudi evidence showing that provider communication and shorter waiting time are important contributors to ED satisfaction [10, 21]. They also align with international ED studies in which respectful provider attitudes, clear information, empathy, and communication with physicians and nurses were repeatedly associated with better patient experience [1, 5, 13, 16]. Waiting time also remained independently associated with overall satisfaction in our model, supporting previous evidence that time spent waiting, and patients’ perceptions of delays, can strongly influence ED satisfaction [17, 18, 22–24].

Not all findings in our study matched earlier reports. Alhabdan et al. found that knowledge of the triage system was associated with higher satisfaction among Saudi patients in the ED [21], whereas no such association was observed in our sample. This difference may reflect variations in patient expectations across settings, and for many patients in the present context, procedural understanding may have been less relevant than interpersonal communication, particularly during acute and stressful encounters.

Other studies have emphasized different domains. Bouaiti et al. identified pain management as the key predictor of satisfaction in their respective settings [25], while Liang et al. found that cleanliness was one of the strongest determinants in China [20]. These elements were less influential in our adjusted analysis, which suggests that satisfaction is shaped by a combination of universal expectations, such as communication, as well as context-specific priorities that differ between populations and healthcare systems. Despite these variations, a consistent theme has emerged in the literature and is reinforced by our findings: interpersonal interaction is central to the patient experience. Regardless of operational or environmental conditions, the ways in which patients are spoken to, supported, and reassured are defining components of ED satisfaction. Our regression results are also in line with findings from Portugal, where satisfaction with doctors and fulfilment of expectations were identified as key predictors of perceived ED care and quality [26].

### Implications for practice

The findings of this study indicate several practical measures that may improve the patient experience in the ED. First, strengthening provider–patient communication should be a priority. Simple behaviors such as clarifying the next steps in care, ensuring patient understanding, and acknowledging delays can meaningfully influence how patients perceive their ED encounter. Second, maintaining transparency regarding waiting times may help to reduce patient anxiety and frustration. Even when delays are unavoidable, consistent updates can improve perceived fairness and trust. Third, improvements to operational workflows, including triage efficiency and mitigation of service bottlenecks, may help to reduce waiting times, which are among the strongest predictors of satisfaction. Collectively, these strategies may support more patient-centered emergency care.

### Strengths and limitations

This study has several strengths. A structured questionnaire was used to capture multiple patient-perceived domains of the ED experience, including provider communication and treatment explanation, administrative processes, waiting-time perceptions, privacy, discharge information, perceived improvement, treatment received, and environmental factors. In addition, the sample size was comparable to that of previous research in this field. The analysis also extended beyond bivariate correlations by incorporating a multivariable regression model, which enabled the identification of independent predictors of overall satisfaction after adjustment for demographic and satisfaction-related variables.

However, several limitations should be noted. The study was conducted at a single tertiary center, which limits the generalizability of the findings to other ED settings. The use of non-probability convenience sampling may have introduced selection bias, although data were collected across multiple shifts to improve the representativeness of the sample. Although the response rate among eligible approached participants was high, the overall proportion of completed surveys relative to all ED attendees during the data-collection shifts was modest because many patients were excluded for clinical reasons, including high-acuity triage levels, arrival by ambulance, medical instability, or impaired cognitive or mental status.

Another limitation is that the questionnaire assessed patient-reported satisfaction rather than objective clinical quality. Therefore, clinical care was measured through patient-perceived items such as provider communication, treatment explanation, perceived improvement, treatment received, discharge information, and privacy. The study did not include objective clinical or operational indicators such as chief complaint, pain score, door-to-provider time, analgesia timeliness, length of stay, diagnostic accuracy, adverse events, or 72-hour revisits. The absence of chief complaint data also limited interpretation because patients with different presentations, such as pain versus non-pain complaints, may prioritize different aspects of ED care. Therefore, the findings should be interpreted as reflecting patient experience rather than direct measurement of clinical performance.

Finally, although the questionnaire was reviewed for content and face validity and demonstrated acceptable internal consistency during pilot testing, it was not evaluated using more formal psychometric methods such as factor analysis. Future studies should validate the tool more rigorously, include multiple ED settings, and combine satisfaction measures with objective clinical and operational indicators.

## Conclusions

This cross-sectional study of 298 eligible ED patients found generally high overall satisfaction (mean 4.24 ± 1.12). After adjustment for demographic and satisfaction-related factors, physician communication (β = 0.305; 95% CI, 0.159 to 0.451; *p* < 0.0001), nurse communication (β = 0.252; 95% CI, 0.118 to 0.385; *p* = 0.0003), explanation of treatment (β = 0.176; 95% CI, 0.053 to 0.298; *p* = 0.0051), and waiting time during stay (β = 0.151; 95% CI, 0.084 to 0.218; *p* < 0.0001) were independent predictors of overall satisfaction. These findings suggest that quality-improvement initiatives in similar tertiary ED settings should prioritize structured provider–patient communication, clear treatment explanations, regular updates about delays, and workflow optimization to reduce waiting-time dissatisfaction. Future multicenter studies should combine patient-reported satisfaction with objective clinical and operational indicators, including chief complaint, acuity level, pain score, door-to-provider time, length of stay, analgesia timeliness, and 72-hour revisits.

## Supplementary Information

Below is the link to the electronic supplementary material.

Supplementary Material 1

Supplementary Material 2

## Data Availability

The datasets used or analyzed in this study are available from the corresponding author on reasonable request.
