# Supplementary material for: Key factors influencing patient satisfaction in the emergency department of a tertiary hospital in Saudi Arabia: a cross-sectional study
Source: BMC Health Serv Res. 2026 Jul 10;26:942. doi: 10.1186/s12913-026-15082-0 (PMC13355336; doi:10.1186/s12913-026-15082-0)
Supplement: Supplementary file 1 — Supplementary Material 1 [file 12913_2026_15082_MOESM1_ESM.pdf]

King Abdullah International Medical Research Center
KAIMRC
APPROVED
IRB
Institutional Review Board

The questionnaire contains **three sheets**. The sheets are as follows:

- **Sheet one:** a consent form for participants to fill out.

- **Sheet two:** contains **five** questions regarding demographic data about the participants, which are:
1. Age
2. Gender
3. Education level
4. Marital status
5. Knowledge about triage

- **Sheet three**: contains a 5-Likert scale questionnaire containing 24 questions to assess patient
Satisfaction levels in the emergency department. The five options that the participant can choose to answer are **(5) Very satisfied, (4)Satisfied, (3)Neutral, (2)Dissatisfied, (1)Very dissatisfied**. The questions are as follows:

- Mode of arrival to the emergency department:

1. How satisfied are you with the directions given by road signs?
2. How satisfied are you with the directions to the parking slots?
3. How satisfied are you with the availability of parking slots allocated for patients visiting the emergency department?

- Administrative skills:

4. How satisfied are you with the check-in procedure by administrative staff?
5. How satisfied are you with the administrative staff's attitude?
6. How satisfied are you with the estimated waiting time given by administrative staff?

- Reception/waiting area:

7. How satisfied are you with the seats in the waiting room?
8. How satisfied are you with the availability of food and beverages in the waiting room?
9. How satisfied are you with the Wi-Fi connection provided by the emergency Department during your stay?
10. How satisfied are you with the availability of restrooms in the emergency department?
11. How satisfied are you with the ventilation (AC) in the waiting room?
12. How satisfied are you with the cleanliness of the waiting room?
13. How satisfied are you with time-delay updates given by hospital staff?

King Abdullah International Medical Research Center
KAIMRC
APPROVED
IRB
Institutional Review Board

14. How satisfied are you with the waiting time?

- <u>Intervention and provider's communication skills:</u>

15. How satisfied are you with the attitude and communication skills of the doctors?
16. How satisfied are you with the attitude and communication skills of the nurses?
17. How satisfied are you with the explanation of the procedure/treatment done on you?
18. How satisfied are you with the improvement of your condition/illness?
19. How satisfied are you with the information/documents provided regarding your health status after the treatment/before leaving?
20. How satisfied are you with management provided by the health staff?
21. How satisfied are you with the privacy provided by health staff during your stay?

- <u>Overall satisfaction:</u>

22. Overall, the quality of care met my expectations.
23. Overall, I am satisfied with my experience in the emergency department.
24. I would recommend this hospital's emergency department to my family and friends.

King Abdullah International Medical Research Center
KAIMRC
APPROVED
IRB
Institutional Review Board

SP24J/021/03

Validated Questionnaire – Arabic Version

الورقة الثانية : بيانات المرضى :

1) **العمر:**

.... (سؤال مفتوح) ....

2) **الجنس :**

- ذكر
- أنثى

3) **المستوى التعليمي :**

- أقل من شهادة الثانوية العامة
- الثانوية العامة
- درجة البكالوريوس
- درجة الماجستير أو أعلى
- أميّ

4) **الحالة الاجتماعية :**

- أعزب
- متزوج
- منفصل
- أرمل/ة

5) **هل تعرف معنى مصطلح " نظام الفرز الطبي الإسعافي (Triage) " ؟**

- لم أسمع به من قبل
- أعرف عنه بعض المعلومات، لكنها ليست كافية
- نعم ، أنا أعرف معنى هذا المصطلح

King Abdullah International Medical Research Center
KAIMRC
APPROVED
IRB
Institutional Review Board

الورقة الثالثة : عوامل رضى المرضى : (مقياس 5-ليكرت)

الوصول إلى قسم الطوارئ :

1. ما مدى رضاك عن التعليمات المعطاة من قبل اللوحات الإرشادية على الطريق ؟
   - راض جدا
   - راض
   - حيادي
   - غير راض
   - غير راض أبدا

2. ما مدى رضاك عن التعليمات المعطاة لمكان مو اقف السيارات ؟
   - راض جدا
   - راض
   - حيادي
   - غير راض
   - غير راض أبدا

3. ما مدى رضاك عن توفر مو اقف سيارات للمرضى القادمين لقسم الطوارئ ؟
   - راض جدا
   - راض
   - حيادي
   - غير راض
   - غير راض أبدا

مهارات التسجيل :

4. ما مدى رضاك عن إجراءات التسجيل والدخول من قبل موظفي الاستقبال ؟
   - راض جدا
   - راض
   - حيادي
   - غير راض
   - غير راض أبدا

5. **ما مدى رضاك عن سلوك موظفي الاستقبال تجاهكم ؟**

- راض جدا
- راض
- حيادي
- غير راض
- غير راض أبدا

6. **ما مدى رضاك عن وقت الانتظار المقدر من قبل موظفي الاستقبال ؟**

- راض جدا
- راض
- حيادي
- غير راض
- غير راض أبدا

<u>منطقة الاستقبال / الانتظار:</u>

7. **ما مدى رضاك عن المقاعد الموجودة في غرفة الانتظار؟**

- راض جدا
- راض
- حيادي
- غير راض
- غير راض أبدا

8. **ما مدى رضاك عن توفر الأطعمة والمشروبات في غرفة الانتظار؟**

- راض جدا
- راض
- حيادي
- غير راض
- غير راض أبدا

9. ما مدى رضاك عن خدمة الانترنت (الواي فاي) المقدمة من قسم الطوارئ ؟

- راض جدا
- راض
- حيادي
- غير راض
- غير راض أبدا

10. ما مدى رضاك عن توفر دورات المياه في غرفة الانتظار؟

- راض جدا
- راض
- حيادي
- غير راض
- غير راض أبدا

11. ما مدى رضاك عن التكييف الموجود في غرفة الانتظار؟

- راض جدا
- راض
- حيادي
- غير راض
- غير راض أبدا

12. ما مدى رضاك عن نظافة غرفة الانتظار؟

- راض جدا
- راض
- حيادي
- غير راض
- غير راض أبدا

13. **ما مدى رضاك عن إبلاغكم بزيادة في وقت الانتظار من قبل موظفي المستشفى ؟**

- راض جدا
- راض
- حيادي
- غير راض
- غير راض أبدا

14. **ما مدى رضاك عن وقت الانتظار؟**

- راض جدا
- راض
- حيادي
- غير راض
- غير راض أبدا

التدخل الطبي و سلوك الممارس الصحي :

15. **ما مدى رضاك عن مهارات تواصل و سلوك الأطباء ؟**

- راض جدا
- راض
- حيادي
- غير راض
- غير راض أبدا

16. **ما مدى رضاك عن مهارات تواصل و سلوك الممرضات ؟**

- راض جدا
- راض
- حيادي
- غير راض
- غير راض أبدا

17. ما مدى رضاك عن شرح الإجراء / العلاج المقدم لك ؟

- راض جدا
- راض
- حيادي
- غير راض
- غير راض أبدا

18. ما مدى رضاك عن تحسن حالتك / مرضك ؟

- راض جدا
- راض
- حيادي
- غير راض
- غير راض أبدا

19. ما مدى رضاك عن المعلومات والمستندات المقدمة لك بخصوص حالتك الصحية بعد العلاج / قبل المغادرة ؟

- راض جدا
- راض
- حيادي
- غير راض
- غير راض أبدا

20. ما مدى رضاك عن العلاج المقدم من الطاقم الصحي ؟

- راض جدا
- راض
- حيادي
- غير راض
- غير راض أبدا

21. ما مدى رضاك عن الخصوصية المقدمة لك أثناء إقامتك في قسم الطوارئ ؟

- راض جدا
- راض
- حيادي
- غير راض
- غير راض أبدا

King Abdullah International Medical Research Center
KAIMRC
APPROVED
IRB
Institutional Review Board

<u>الرضى العام :</u>

22. **إجمالا ، كانت جودة الرعاية الطبية المقدمة لي مطابقة لتوقعاتي .**
    - أوافق بشدة
    - أوافق
    - محايد
    - لا أوافق
    - لا أوافق بشدة

23. **إجمالا ، أنا راض عن تجربتي في قسم الطوارئ .**
    - أوافق بشدة
    - أوافق
    - محايد
    - لا أوافق
    - لا أوافق بشدة

24. **سأوصي بقسم الطوارئ في هذا المستشفى لعائلتي وأصدقائي .**
    - أوافق بشدة
    - أوافق
    - محايد
    - لا أوافق
    - لا أوافق بشدة
