# Supplementary material for: Key factors influencing patient satisfaction in the emergency department of a tertiary hospital in Saudi Arabia: a cross-sectional study
Source: BMC Health Serv Res. 2026 Jul 10;26:942. doi: 10.1186/s12913-026-15082-0 (PMC13355336; doi:10.1186/s12913-026-15082-0)
Supplement: Supplementary file 2 — Supplementary Material 2 [file 12913_2026_15082_MOESM2_ESM.pdf]

Full Factor Correlation Matric

	Age	Gender	Education	Marital Status	Triage	directions of road	parking	registration process	regestration manners	estimated time of regrst	seats	foods and drinks	bathroom	a/c	clean	time change	waiting time in waiting room	comm doctors	comm nurses	tx explain	improvement	info provided and booklet	treatment	privacy
Age	1.0000	-0.0503	-0.5416	0.5063	-0.2079	0.0654	0.0016	0.0282	-0.0129	-0.1024	-0.0461	0.0118	0.0167	-0.0983	-0.0630	-0.0702	0.0181	-0.0361	-0.0169	-0.0004	0.0043	-0.1792	-0.0430	-0.0080
Gender	-0.0503	1.0000	-0.1140	0.3334	-0.0863	0.1229	0.0221	-0.0063	0.0031	0.0294	0.0483	0.0659	0.0394	0.0579	0.0367	0.0995	-0.0142	-0.0004	-0.0345	0.0259	-0.0136	-0.0968	-0.0334	-0.0235
Education	-0.5416	-0.1140	1.0000	-0.4155	0.2399	-0.0337	0.0085	0.0206	0.0413	0.0738	0.0650	0.0190	0.0605	0.0662	0.0762	0.0483	-0.0683	0.0253	-0.0042	0.0461	0.0383	0.1703	0.0884	0.0389
Marital Status	0.5063	0.3334	-0.4155	1.0000	-0.1402	0.2228	0.0500	0.0330	0.0336	-0.0472	0.0280	0.0444	-0.0204	-0.0898	-0.0161	-0.0533	-0.0297	0.0098	-0.0186	0.0200	-0.0614	-0.1406	-0.0773	0.0116
Triage	-0.2079	-0.0863	0.2399	-0.1402	1.0000	0.0058	0.0348	-0.0255	-0.0456	0.1547	-0.0128	-0.1682	0.0045	-0.0090	0.0003	0.0161	0.0102	0.0609	0.0391	-0.0692	-0.0916	0.1150	-0.0401	-0.0093
directions of road	0.0654	0.1229	-0.0337	0.2228	0.0058	1.0000	0.2451	0.2408	0.2119	0.0266	0.1932	0.1526	0.1866	0.0224	0.1040	-0.0289	0.1441	0.2243	0.0712	0.1927	0.1304	0.0785	0.1412	0.1073
parking	0.0016	0.0221	0.0085	0.0500	0.0348	0.2451	1.0000	0.0421	0.0167	0.1437	0.0137	-0.0725	-0.0078	-0.0565	-0.0990	0.0283	0.1138	0.0431	0.0116	0.0833	0.1060	0.1162	0.1426	0.0365
registration process	0.0282	-0.0063	0.0206	0.0330	-0.0255	0.2408	0.0421	1.0000	0.8601	0.1663	0.2605	0.1839	0.1811	0.2232	0.2113	0.1147	0.2081	0.1863	0.1515	0.1565	0.1234	0.0802	0.1262	0.1361
regestration manners	-0.0129	0.0031	0.0413	0.0336	-0.0456	0.2119	0.0167	0.8601	1.0000	0.1864	0.2743	0.2028	0.1953	0.2288	0.2276	0.1715	0.2072	0.2901	0.2014	0.2625	0.1645	0.1416	0.1828	0.1871
estimated time of regrst	-0.1024	0.0294	0.0738	-0.0472	0.1547	0.0266	0.1437	0.1663	0.1864	1.0000	0.1648	0.0881	0.0364	0.1488	0.1651	0.4752	0.4352	0.1363	0.1600	0.1029	0.1223	0.2037	0.1681	0.1370
seats	-0.0461	0.0483	0.0650	0.0280	-0.0128	0.1932	0.0137	0.2605	0.2743	0.1648	1.0000	0.5143	0.5672	0.5979	0.6099	0.1666	0.2121	0.2298	0.1699	0.1882	0.0878	0.1326	0.1324	0.1358
foods and drinks	0.0118	0.0659	0.0190	0.0444	-0.1682	0.1526	-0.0725	0.1839	0.2028	0.0881	0.5143	1.0000	0.6396	0.4911	0.5102	0.1275	0.1012	0.1416	0.1500	0.1972	0.0764	0.0359	0.0527	0.0873
bathroom	0.0167	0.0394	0.0605	-0.0204	0.0045	0.1866	-0.0078	0.1811	0.1953	0.0364	0.5672	0.6396	1.0000	0.5684	0.6421	0.0837	0.0901	0.1268	0.1478	0.2166	0.1542	0.0835	0.1352	0.1602
a/c	-0.0983	0.0579	0.0662	-0.0898	-0.0090	0.0224	-0.0565	0.2232	0.2288	0.1488	0.5979	0.4911	0.5684	1.0000	0.6790	0.1829	0.1911	0.1450	0.1193	0.1907	0.1979	0.0789	0.1852	0.0861
clean	-0.0630	0.0367	0.0762	-0.0161	0.0003	0.1040	-0.0990	0.2113	0.2276	0.1651	0.6099	0.5102	0.6421	0.6790	1.0000	0.1614	0.1058	0.1091	0.0480	0.2053	0.1287	0.1275	0.1267	0.1531
time change	-0.0702	0.0995	0.0483	-0.0533	0.0161	-0.0289	0.0283	0.1147	0.1715	0.4752	0.1666	0.1275	0.0837	0.1829	0.1614	1.0000	0.4231	0.1725	0.0957	0.0817	0.1428	0.1563	0.1570	0.1047
waiting time in waiting room	0.0181	-0.0142	-0.0683	-0.0297	0.0102	0.1441	0.1138	0.2081	0.2072	0.4352	0.2121	0.1012	0.0901	0.1911	0.1058	0.4231	1.0000	0.2269	0.1872	0.1536	0.1311	0.0346	0.1411	0.1615
comm doctors	-0.0361	-0.0004	0.0253	0.0098	0.0609	0.2243	0.0431	0.1863	0.2901	0.1363	0.2298	0.1416	0.1268	0.1450	0.1091	0.1725	0.2269	1.0000	0.5680	0.4516	0.2795	0.1981	0.2996	0.1933
comm nurses	-0.0169	-0.0345	-0.0042	-0.0186	0.0391	0.0712	0.0116	0.1515	0.2014	0.1600	0.1699	0.1500	0.1478	0.1193	0.0480	0.0957	0.1872	0.5680	1.0000	0.1737	0.1188	0.0757	0.1354	0.2240
tx explain	-0.0004	0.0259	0.0461	0.0200	-0.0692	0.1927	0.0833	0.1565	0.2625	0.1029	0.1882	0.1972	0.2166	0.1907	0.2053	0.0817	0.1536	0.4516	0.1737	1.0000	0.3715	0.2946	0.3601	0.2583
improvement	0.0043	-0.0136	0.0383	-0.0614	-0.0916	0.1304	0.1060	0.1234	0.1645	0.1223	0.0878	0.0764	0.1542	0.1979	0.1287	0.1428	0.1311	0.2795	0.1188	0.3715	1.0000	0.3747	0.9295	0.1160
info provided and booklet	-0.1792	-0.0968	0.1703	-0.1406	0.1150	0.0785	0.1162	0.0802	0.1416	0.2037	0.1326	0.0359	0.0835	0.0789	0.1275	0.1563	0.0346	0.1981	0.0757	0.2946	0.3747	1.0000	0.4236	0.0932
treatment	-0.0430	-0.0334	0.0884	-0.0773	-0.0401	0.1412	0.1426	0.1262	0.1828	0.1681	0.1324	0.0527	0.1352	0.1852	0.1267	0.1570	0.1411	0.2996	0.1354	0.3601	0.9295	0.4236	1.0000	0.1204
privacy	-0.0080	-0.0235	0.0389	0.0116	-0.0093	0.1073	0.0365	0.1361	0.1871	0.1370	0.1358	0.0873	0.1602	0.0861	0.1531	0.1047	0.1615	0.1933	0.2240	0.2583	0.1160	0.0932	0.1204	1.0000
